# What values drive communities’ nutrition priorities in a resource constrained urban area in South Africa?

**DOI:** 10.1186/s12889-023-15761-1

**Published:** 2023-05-12

**Authors:** Agnes Erzse, Teurai Rwafa-Ponela, Susan Goldstein, Molebogeng Motlhatlhedi, Daniella Watson, Karen J. Hofman, Marion Danis, Shane A. Norris, Kate A. Ward, Aviva Tugendhaft, on behalf of the INPreP study group, Abraham Oduro, Adélaïde Compaoré, Aminata Welgo, Caroline Fall, Cornelius Debpuur, Doreen Ayibisah, Edith Dambayi, Engelbert Nonterah, Esmond W. Nonterah, Halidou Tinto, Hermann Sorgho, James Adoctor, Josephine Addi, Kadija Ouedraogo, Karim Derra, Keith Godfrey, Marie-Louise Newell, Mark Hanson, Mary Barker, Maxwell Dalaba, Michael Banseh, Palwendé R. Boua, Paul Welaga, Paula Beeri, Polly Hardy-Johnson, Samuel Chatio, Sarah Kehoe, Stephanie Wrottesley, Winfred Ofosu

**Affiliations:** 1 SAMRC/ Wits Centre for Health Economics and Decision Science, PRICELESS SA, Faculty of Health Sciences, School of Public Health, University of Witwatersrand, Johannesburg, South Africa; 2 SAMRC/ Wits Developmental Pathways for Health Research Unit, Faculty of Health Sciences, School of Clinical Medicine, University of the Witwatersrand, Johannesburg, South Africa; 3 Global Health Research Institute, School of Human Development and Health, Faculty of Medicine, University of Southampton, Southampton, UK; 4 Department of Bioethics, National Institutes of Health, Bethesda, MD USA; 5 Medical Research Council Lifecourse Epidemiology Centre, University of Southampton, Southampton, UK

**Keywords:** Decision-making, Malnutrition, Maternal-child health services, Resource allocation, Community involvement, Social values

## Abstract

**Background:**

Voices of under-resourced communities are recognised as important yet are often unheard in decisions about healthcare resource allocation. Deliberative public engagement can serve as an effective mechanism for involving communities in establishing nutrition priorities. This study sought to identify the priorities of community members of a South African township, Soweto, and describe the underlying values driving their prioritisation process, to improve nutrition in the first 1000 days of life.

**Methods:**

We engaged 54 community members (28 men and 26 women aged > 18 years) from Soweto. We conducted seven group discussions to determine how to allocate limited resources for prioritising nutrition interventions. We used a modified public engagement tool: CHAT (Choosing All Together) which presented 14 nutrition intervention options and their respective costs. Participants deliberated and collectively determined their nutritional priorities. Choices were captured quantitatively, while group discussions were audio-recorded. A thematic analysis was undertaken to identify the reasons and values associated with the selected priorities.

**Results:**

All groups demonstrated a preference to allocate scarce resources towards three priority interventions—school breakfast provisioning, six-months paid maternity leave, and improved food safety. All but one group selected community gardens and clubs, and five groups prioritised decreasing the price of healthy food and receiving job search assistance. Participants’ allocative decisions were guided by several values implicit in their choices, such as fairness and equity, efficiency, social justice, financial resilience, relational solidarity, and human development, with a strong focus on children. Priority interventions were deemed critical to supporting children’s optimal development and well-being, interrupting the intergenerational cycle of poverty and poor human development in the community.

**Conclusion:**

Our study demonstrates how public engagement can facilitate the incorporation of community values and programmatic preferences into nutrition priority setting, enabling a responsive approach to local community needs, especially in resource constrained contexts. Findings could guide policy makers to facilitate more appropriate decisions and to improve nutrition in the first 1000 days of life.

**Supplementary Information:**

The online version contains supplementary material available at 10.1186/s12889-023-15761-1.

## Background

Maternal and child malnutrition remains a global problem with significant implications for survival rates, children’s cognitive and physical development, and economic productivity in adulthood [1]. Globally, 37% of pregnant women were affected by anaemia in 2019, 10% of women of reproductive age were underweight, and 35% were overweight in 2016 [2]. The poor nutritional status of a woman before and during pregnancy and lactation is of great concern, given its vital role in determining the mother’s health and that of her offspring [3, 4]. Globally, an estimated 149 million children under the age of 5 were stunted (too short for age), 45 million were wasted (too thin for height), and 38.9 million were overweight or obese in 2020 [5]. Around 45% of deaths among children under 5 years of age are linked to undernutrition, with the majority occurring in low- and middle-income countries [5], including South Africa. Almost a third (31%) of South African children who die in hospitals are severely malnourished [6], and 14% are born with low birth weight [7]. If these children survive, they are more likely to be stunted, which continues to be the most prevalent form of childhood malnutrition (21%), followed by overweight and obesity (12%) [4]. Obesity (33%) [8] and anaemia (31%) [9] in pregnant women are significant contributors to child malnutrition in South Africa [10, 11].

The first 1000 days of life, from conception to a child’s second birthday, are vital in preventing malnutrition and breaking the intergenerational cycles of stunting, underweight and obesity. Early life stages play a pivotal role in establishing the foundation for optimum health and development [12]. As illustrated by the Developmental Origins of Health and Disease concept [13], the first 1000 days of life is a window of opportunity to efficaciously intervene to reduce exposure to adverse factors and prevent adult diseases. Despite having high impact interventions directed at the first 1000 days [14], South Africa’s limited progress towards achieving global 2025 nutrition targets [7] suggests a gap between policy, implementation and practice. Accelerating progress and necessary investments for nutrition is a challenging task for South African policy makers, given the country’s multiple disease burdens and reduced resources due to both COVID-19 related public spending and economic slowdowns [15]. The need for evidence-informed resource allocation for nutrition is more urgent than ever.

In any process to determine which service is most important, value judgements are inevitable. Even decisions that appear to be based on “technical” grounds, such as clinical effectiveness and economic efficiency, can be influenced by the values of the decision maker and the norms of the organization making the decision [16]. These judgements are referred to as social value judgements and can be defined as judgments made based on the moral or ethical values of a particular society [17].

As with most health systems globally, since the establishment of democracy, the South African health system embodies several social values that underpin its goals, including consultation with the public [18]. Beyond this, there is a democratic and constitutional commitment to meaningful public participation, which should go beyond consultation to involve citizens more deeply in decisions that affect their health [19]. There is a growing recognition that such democratic commitments warrant more room for public voices in decisions about resource allocation, which should embody the values of those populations served by the healthcare system in question [17]. However, measuring and interpreting societal value judgements for health priority setting remains a challenge [20].

Deliberative public engagement, a process that engages the public in value-based discourse around a specific topic, has been recognised as especially useful in eliciting shared social values among community members. Deliberation relies on mutual understanding, communication, and the sharing of diverse views as a group as opposed to individual preferences driven by self-reflection, features that distinguish deliberations from the aggregate preferences of group members [21].

It is believed that well-designed and executed public deliberation that allows effective interaction among participants that results in social learning and the development of shared meanings and values can enhance the legitimacy and acceptability of policy decisions. This results in more appropriate, feasible, inclusive, and just recommendations. Public deliberation may also increase public buy-in and trust in governing institutions [22, 23].

In South Africa, policy decisions to prevent and manage mother and child malnutrition have been largely driven by policy makers and based on historical funding, the burden of disease assessments and, more recently, economic considerations [24]. Meaningful public participation as entrenched in South Africa’s Constitution [19] and the *National Department of Health Strategic Plan* [18] has received less attention. The aim of this paper was to identify and describe the priorities of members of an urban township community in Soweto, Johannesburg, on how to improve the nutrition of mothers and children and the values driving their selected priorities.

## Methods

To facilitate public deliberation about nutrition priorities, we modified a public engagement tool, CHAT (Choosing All Together) [25]. CHAT is a simulation exercise to engage the public in decision-making, originally designed for health care priority setting. CHAT comprises various rounds of deliberation where participants work individually and then as a group to distribute a limited number of resources on a board as they select from a wide range of intervention options. CHAT and the formative work for creating the tool were part of a NIHR-funded study, ‘Improved Nutrition Preconception Pregnancy Post-Delivery’ (INPreP).

### Modifying CHAT to nutrition priority setting

To adapt CHAT to setting priorities for maternal and child nutrition spending, we followed the steps outlined in Tugendhaft et al. (2021) [26]. The modification process was informed by policy and literature reviews, interviews with policy makers and group discussions with community members [27].

We synthesised information from the various data sources and developed a series of interventions that captured existing and ‘new’ potential interventions to improve maternal and child nutrition. The result was 14 possible interventions designed to reflect realistic options that addressed the needs of communities in Soweto [27]. Five interventions were nutrition-specific, influenced the immediate causes of malnutrition and were delivered through the healthcare system (see interventions in Table 3 marked with “*”). Nine interventions were nutrition-sensitive, addressed the underlying determinants of nutrition [1], and were situated in non-health sectors (interventions in Table 3 without “*”). Researchers from the broader INPreP group reviewed each intervention. A costing exercise using secondary data sources was undertaken to estimate the relative cost of each intervention to present credible cost estimates. We relied on an ingredient-based, bottom-up costing approach to assess the amount of each ingredient (input) required to implement an intervention and assigned costs accordingly to generate aggregate costs [28]. Costing included both program and individual-related costs. For Soweto-specific population estimates and epidemiological parameters, we used 2011 national census data [29] and searched the literature on Soweto. We relied on district-level policy documents for other parameters, such as the number of schools, community centres, and antenatal clinics. Cost components and quantity assumptions were developed by researching existing programs offering similar interventions. Prices and unit costs of the components were collected from various sources, including national databases such as the Department of Public Service and Administration Salary Scale 2021 [30] and international databases such as the WHO-CHOICE [31]. All prices were in 2021 South African Rand (ZAR), and the total cost of the interventions were converted into sticker amounts for the exercise based on an existing actuarial model developed for previous CHAT exercises (1 sticker = 0.5% of total cost) [26].

**Fig. 1:**
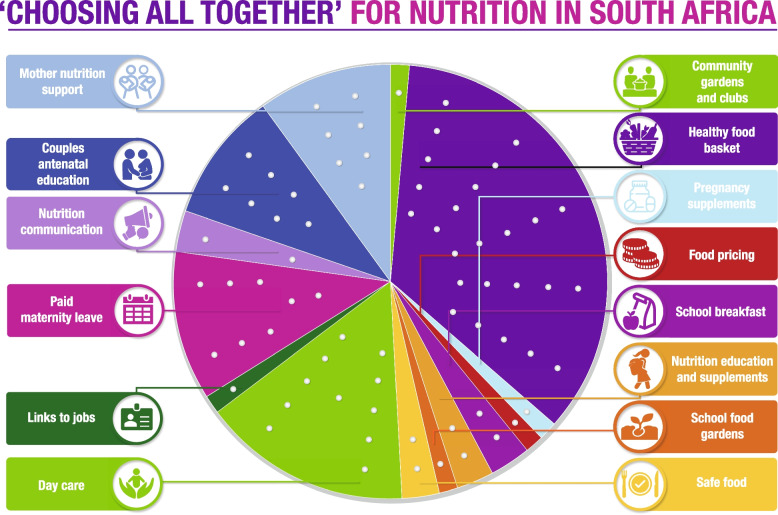
Soweto CHAT board with 14 interventions to improve mother and child nutrition. Adapted from the original CHAT board as in Goold et al. (2005) [25]

The result of this process is displayed on the CHAT board shown in Fig. 1. Intervention options are depicted as slices of the pie. At the time of the study, the total cost of interventions was ZAR 136 million, ZAR 1270 per person (USD 8 million in total and USD 77 per person), represented by 70 open sticker holes. Participants were given a limited budget of 42 stickers (60% of the total) to allocate accordingly across these intervention options. They could either select an intervention in its entirety by allocating the required stickers (‘price-tag’) or choose to forgo an intervention. Participants received an information booklet describing interventions, associated icons, and sticker values, written in plain English and designed to be credible and comprehensible to a lay audience. Before the CHAT exercise, the booklet was refined with the help of qualitative researchers with extensive previous research experience in the community and a deep understanding of the type of language (i.e., wording) communities would be most familiar with. A detailed description of each intervention is provided in Additional file 1.

Participants allocated stickers in two rounds. In the first round, participants worked in pairs. This was a practice round for participants to familiarise themselves with the exercise. It was not documented nor evaluated quantitatively. In the second round, participants set priorities as a group. Before the group round, participants listened to and discussed nutrition scenarios (“events”) that illustrated the consequences of their choices from round 1 (see an example in Table 1). A trained facilitator asked participants to make fair decisions for fellow community members throughout the exercise.

**Table 1:**
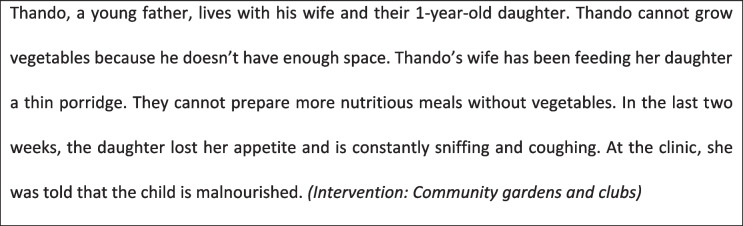
Example of nutrition scenario card for CHAT

### Setting

The study was conducted in Soweto, South Africa's largest township near Johannesburg. Soweto is a predominantly Black, low-income area with approximately 19% of its population earning no income and only 3% earning more than ZAR 307 thousand (USD 19 thousand) a year [29]. The prevalence of nutrition-related diseases, particularly among children and women, is high. In 2019, a sample of non-pregnant women living in Soweto showed that 44% of women were overweight or obese, almost 9% were underweight, and 30% were anaemic [32]. Furthermore, a study conducted in 2016 on a cohort of pregnant women residing in Soweto revealed that 66% of them were overweight or obese at their first antenatal clinic visit. Out of these, 12% were also affected by gestational diabetes [33]. Child malnutrition under the age of 5 years manifests most prominently in the form of high stunting rates, reaching a peak at two years of age at over 34% for black boys and overweight and obesity rates at around 24% for black girls [34]. All study procedures occurred in September 2021 in the SAMRC/Wits Developmental Pathways for Health Research Unit (DPHRU) within the precinct of Chris Hani Baragwanath Academic Hospital, a tertiary care hospital in Soweto.

### Sampling and recruitment

Community recruiters purposely selected participants. Community members were approached to participate in a group discussion, initially in person and followed up telephonically. These participants were then asked to suggest other potential participants they thought would be willing to participate, and they were then contacted telephonically. Participants were eligible to participate if they lived in Soweto, were aged ≥ 18 years and cared for a child. Participants were informed about the study objectives, and only those who voluntarily agreed to participate signed a consent form. Participants were grouped into one of five groups: older women (aged 50 + years), younger women (aged 18–49 years), older men (aged 50 + years), younger men (18–49 years), and both men and women (18 + years). Different age and gender groups were selected to cover various stages of the reproductive life course and to avoid older participants from potentially inhibiting younger participants' expressions.

### Data collection

Group discussions were held at the research centre in Soweto. Following COVID-19 safety protocols, the discussions took place in a private meeting room to avoid disturbance and to create a comfortable atmosphere for participants. The discussions were conducted by a multilingual facilitator and two research assistants. Before group discussions, we brought together the facilitator and researchers for two days of training on CHAT. The facilitator and researchers were trained on principles of priority setting, CHAT materials, encouraging dialogue, probing, and eliciting reasons for each group choice.

All participants completed a short self-administered demographic questionnaire before CHAT. A detailed facilitator script was used to guide the discussions. Two forms of data were collected to capture participants’ preferences for allocating funds for nutrition interventions. First, we recorded the outcome of the deliberation process, which was the selection of nutrition interventions by the groups. Second, we collected data on the reasoning behind participants’ choices. The feasibility of our data collection method was verified by conducting a pilot group discussion. Results from the pilot were included in the analysis. The discussions were conducted in English (as all participants could speak English); however, they were encouraged to express themselves in their vernacular languages (Xhosa, Zulu or Sotho). All CHAT materials were in English, and the two research assistants were available during the discussions if participants needed translation. They also assisted with note-taking of discussions, which lasted between 2.5 to 3 h. These were audio-recorded, transcribed, and translated into English, where necessary. All participants were reimbursed for transport to the research centre and were provided refreshments during the discussions.

### Data analysis

Participant socio-demographic characteristics and the selection frequencies for each intervention were analysed using descriptive statistics in MS Excel. Qualitative data was analysed thematically, following Strauss and Corbin’s method of open, axial and selective codes [35]. Two researchers (AE and TR) independently coded two transcripts line by line to develop an initial list of codes (open coding). A process of constant comparison was employed whereby subsequent transcripts were coded using this list, and new themes which emerged from further group discussions were added to the list upon consultation with two other researchers in the team (AT and SG). Data analysis was facilitated using MAXQDA software version 2021 to manage transcripts, themes and quotes. Any discrepancies in the coding process were resolved by discussion during monthly research meetings between four authors (AE, TR, AT, and SG). Codes were organised and re-organised into broader categories based on thematic similarities between codes (axial coding). Thereafter, selective coding was conducted to place codes into categories. This was guided by a deductive approach where categories were based on our research questions about the reasons for and against investing in interventions. Two researchers (AT and SG) checked all codes to ensure consistency and consensus on axial and selective codes. Lastly, we examined the underlying assumptions, beliefs, or perspectives upon which the participants’ decisions were based; and called these values. Matching values with codes represented the final step in data analysis. To ensure the appropriateness of the terminologies of values, we consulted public health ethics literature [36, 37], and assigned values to reasons for selection of interventions and reasons against selection of interventions, which resulted in 15 values. A research member (DM) with a bioethics background reviewed values related to codes. Participants' priority interventions, reasons behind the choices, and their associated values were used to structure the results. Reporting of the findings adheres to COREQ guidelines [38].

## Results

### Characteristics of study participants

Fifty-four participants were included in seven group discussions. Among the total sample, 28 (51%) were men, and 26 (49%) were women. Thirty (56%) participants were single, and 16 (30%) were married or partnered. Most participants lived in a mortgaged house (35%), in a single room built as an extension in the backyard of other households (28%) or occupied a shack (13%) or government subsidised house (13%). Regarding the source of income, 21 (81%) women relied only on social grants. Among men, 3 (11%) were formally employed, 15 (54%) were working in informal employment, and 9 (32%) relied on social grants as a source of income. Thirty-five (65%) participants earned less than ZAR 3000 (USD 187) per month. All participants had some education, with 17 (61%) men and 8 (31%) women having completed secondary school. The summary of the participants and their details are provided in Table 2.

**Table 2:** Sociodemographic characteristics of participants in the group discussions conducted in Soweto in 2021

	Men (*n* = 28)		Women (*n* = 26)		Total (*n* = 54)	
**Age (years)**						
Minimum–maximum	21–64		18–63		18–64	
*Mean [SD]*	41.714 [13.6]		44.24 [12.6]		42.9 [13.0]	
**Number of dependent babies and children**	N %		N	%	N	%
*1*	6	21%	6	23%	12	22%
*2–4*	13	46%	10	38%	23	43%
*5 and above*	3	11%	7	27%	10	19%
**Marital status**						
*Single never married*	14	50%	16	62%	30	56%
*Married*	4	14%	3	12%	7	13%
*Partnered*	5	18%	4	15%	9	17%
*Separated*	2	7%	1	4%	3	6%
*Divorced*	3	11%	0	0%	3	6%
*Widowed*	0	0%	2	8%	2	4%
**Highest level of schooling**						
*Primary school*	2	7%	2	8%	4	7%
*Some high school*	7	25%	13	50%	20	37%
*Completed high school*	17	61%	8	31%	25	46%
*Diploma*	2	7%	3	12%	5	9%
**Household income**						
*ZAR 3000 or less*	14	50%	21	80%	35	65%
*ZAR 3001- 10,000*	14	50%	4	15	18	33%
*ZAR 10,001–20,000*	0	0%	1	4%	1	2%
**Source of income**						
*From government grants*	9	32%	21	81%	30	56%
*From employment*	3	11%	2	8%	5	9%
*From grants and employment*	1	4%	1	4%	2	4%
*Other (informal work)*	15	54%	1	4%	16	30%
**Type of house**						
*Mortgaged house*	12	43%	7	27%	19	35%
*Single outside room*	7	25%	8	31%	15	28%
*Government subsidy, RDP house*	2	7%	5	19%	7	13%
*Shack (informal house, commonly made of tin)*	4	14%	3	12%	7	13%
*Single room inside a house*	2	7%	2	8%	4	7%
*Other*	1	4%	1	4%	2	4%

### Priority choices

Table 3 shows interventions prioritised by the groups, with 1 indicating that an intervention was selected and 0 meaning it was not, as well as the associated values underlying these choices. Three interventions were chosen by all groups, these were paid maternity leave, school breakfast, and safe foods. All but one group selected community gardens and clubs, and 5 out of 7 groups chose food pricing and links to jobs. Dominant values underlying participants’ allocative decisions included financial resilience, solidarity, food safety and security, mental well-being, equity, preventing harm, communitarianism and capability with a strong focus on children.

**Table 3:** Allocation decisions of participants and associated values

Intervention	G0	G1	G2	G3	G4	G5	G6	Total	Associated values
**Paid maternity leave**	1	1	1	1	1	1	1	7	*Pro-* Autonomy, Financial resilience, Capability, Mental well-being; *Pro & con –* Solidarity; *Con*—Efficiency
**School breakfast**	1	1	1	1	1	1	1	7	*Pro-* Financial resilience, Capability, Solidarity, Food security, Mental well-being
**Safe food**	1	1	1	1	1	1	1	7	*Pro-*, Food safety, Capability; *Pro & con –* Communitarianism
**Community gardens and clubs**	1	1	1	1	1	1	0	6	*Pro—*Communitarianism, Financial resilience, Equity, Capability, Preventing harm, Solidarity, Social cohesion, Food security
**Food pricing**	1	0	1	0	1	1	1	5	*Pro—*Solidarity, Equity, Food security, Efficiency, Necessity
**Links to jobs**	1	0	1	1	1	1	0	5	*Pro –* Necessity, Capability, Financial resilience, Preventing harm, Food security, Mental well-being; *Con –* Communitarianism
**Nutrition communication**^a^	0	1	1	0	0	1	1	4	*Pro—*Equity, Capability
**School food gardens**	1	1	0	1	1	0	0	4	*Pro—*Communitarianism, Financial resilience, Food security, Solidarity, Necessity
**Healthy food basket**	0	0	1	1	0	1	1	4	*Pro—*Communitarianism, Financial resilience, Equity, Food security; *Con—*Evidence based, Trust
**Pregnancy supplements**^a^	0	1	0	1	1	0	0	3	*Pro—*Solidarity, Capability, Mental well-being
**Mother nutrition support**^a^	1	1	0	0	1	0	0	3	*Pro—*Solidarity, Capability, Preventing harm
**Daycare**	1	1	0	0	1	0	0	3	*Pro—*Capability, Solidarity, Financial resilience; *Con –* Food safety
Nutrition education and supplements*	1	0	0	0	0	0	1	2	*Pro—*Equity, Solidarity, Capability; *Con—*Necessity
**Couple antenatal education**^a^	0	1	0	0	1	0	0	2	*Pro—*Equity, Autonomy, Social cohesion, Capability, Mental well-being

^a^Indicates a nutrition-specific intervention delivered through the healthcare system. Interventions without “*” were classified as nutrition-sensitive

G0: Pilot group (mixed women and men > 18 years), G1: group 1 (older women > 50 years), G2: group 2 (younger men 18–49 years), G3: group 3 (older men > 50 years), G4: group 4 (younger women 18–49 years), G5: group 5 (mixed women and men > 18 years), G6: group 6 (mixed women and men > 18 years)

Pro & con indicate if the value was used in support of or against choosing an intervention

### Allocation decisions

#### Paid maternity leave

Care for mothers and infants during the postpartum phase was associated with high costs by participants. The provision of money during the first six months postpartum was considered to directly address social and economic precursors of poor nutrition for mothers and children.

On the one hand, paid maternity leave was associated with increased financial resilience. At an individual level, increased income was associated with increased autonomy for mothers to buy baby necessities and nutritious foods when required.

*“She has things she needs. I would not be able to leave work just to buy her bread. I cannot leave work just to buy her apples. If she needs apples, she would have to wait for me to return home at 7 pm. Now, the money that she receives let her use it. It will assist her. It will help both of us.” (Group 2 – younger men, 18-49)*

By ensuring a mother’s economic autonomy, paid maternity leave was also perceived to assist with paying for transportation needs for urgent clinic visits for both mothers and children.

*“If you call an ambulance, it takes time to reach you. However, when money is available, you can send someone to ask the transport person to take you to the hospital in a timely manner. At least, there will be money to pay for that transport.” (Group 2 – younger men, 18-49)*

At household level, availability of paid maternity leave was associated with financial resilience for the whole family by complementing the household’s income and easing strain of relying on one salary.

*“If a person is on maternity leave and is not working, it means there is no income. So as much as we have high unemployment rates, chances are maybe the father does not work […], so if they do not have money while they are on maternity leave, it affects the entire family. So, if ever she is getting paid leave, it eases the burden at home and will be able to take care of the child.” (Group 5 - mixed women and men, 18*+*)*

Safeguarding the initial six months from economic pressures of having to work was considered to lead to several positive health and non-health benefits, including reduced psychological stress associated with financial needs, hunger, and childcare, as well as increased breastfeeding and bonding time, which were perceived to be crucial to protect and support children’s capabilities in terms of health, growth, and development.

While all groups selected paid maternity leave in their final package, there was considerable deliberation around its inclusion based on costs and potential benefits. When faced with their limited CHAT budget, some wished to reallocate money from the already selected paid maternity leave toward mother nutrition support instead. For a participant, the potential benefits of paid maternity leave were not considered an efficient use of resources without increasing mothers’ capabilities—first-time mothers in particular—to care for children and themselves through receiving additional nutrition support. Deliberation highlighted the trade-off between financial resilience and the improved capabilities of mothers.

*“Okay, you will get your maternity leave, but as a new mother, you haven’t got any support, you don’t have any education on how to handle the newborn baby. So, I don’t think it would make any difference if you get that maternity leave money. The most important thing is for a new mother to get all the support that she needs. […] A new mother needs someone who will tell them what to expect. Then again, someone who is still pregnant and going to be a new mother is a person who may drink, or she was drinking; [she] needs the kind of support that would inform her that alcohol is not good for you when you are in this situation. [...] So, I don’t see maternity leave as important as this one.” (Group 2 - younger men, 18-49)*

Nevertheless, the groups decided to retain paid maternity leave as the priority option.

Group deliberations were also driven by solidarity towards young first-time mothers and by various equity considerations, including implications of the intervention for those who are unemployed, those with lower incomes, and the inclusion of men. One participant, for example, challenged Group 6 by advocating for equal inclusion of men in the paid ‘parental’ leave discussion.

*“Even the dads should be included in raising children, so I am not sure if I must be confined to this [intervention] or go further because I believe fathers play a vital role in the upbringing of the child. So paid maternity leave, not only for mothers but for fathers as well.” (Group 6 - mixed women and men, 18*+*)*

Counter arguments to paternity leave focused on a common theme of “irresponsible fatherhood”, with some participants expressing their lack of trust in fathers to use the additional income from the paternity leave for the benefit of mothers and infants.

#### School breakfast

Participants emphasised the need for breakfast for school-going children, specifically those from households struggling with low incomes or unemployment, indicating a sense of group solidarity and equity considerations. School breakfast was perceived to mitigate the adverse effects of household poverty and food insecurity and to ensure equitable inclusion in education of those less privileged.

*“School breakfast, we have many families who cannot afford it and live in poverty. So, you find that children leave their homes without eating. When they arrive at school, if they have not eaten in the morning, there is a lack of concentration by a child. So, if they know that they will receive food when they get to school, it is good for them and their education as well.” (Group 2 - younger men, 18-49)*

Participants who selected school breakfast perceived it as crucial to reducing psychological distress of food insecurity on caregivers and children alike.

*“It will help them have less stress and anxiety about hunger because a lot of the children do not carry pocket money, so they tend to worry about what they will eat. Some of them even fall asleep because they are hungry.” (Group 6 - mixed women and men, 18*+*)*

By selecting school breakfast, participants also wished to enhance children’s capabilities by increasing their focus in school, realising their future potential as individuals and contributing to the betterment of their communities.

*“I just want to add on how it helps the community, you know when you are well fed, you are able to concentrate, which means the children are able to go to school, stay focused, and they can do better in their studies, meaning our own community could produce doctors, teachers. That’s the long term on how the community gets assisted.” (Group 3 - older men, 50*+*)*

Another reason for investing in school breakfast was underpinned by foreseen implications of increased financial resilience of households by saving money on food.

*“When they are at school, we are also able to save on money as the children are at school from the morning until after school in the afternoon. So, the whole time that they are at school, we are happy because we know that the child is fed and has left the house without any food. Sometimes, the money in the home finishes before the end of the month, and there is nothing to eat. But in those instances, you will know that at least when the child is at school, they are being fed.” (Group 1 - older women, 50*+*)*

#### Safe foods

Participants selecting safe foods saw the cause of poor nutrition and health in the community deriving from unhygienic preparation, serving and storing unsafe foods sold on the street and provided to children in schools and crèches. Consequently, they favoured investment inspections and improved food safety among food providers and sellers.

The most common reason for investment in safe foods related to foreseen reduction of children getting sick from contaminated food and increased knowledge of food hygiene amongst children.

*“It'll be very good because it will decrease the number of sick children that we get at the crèches, and the children will also be aware of the food that they’re eating.” (Group 6 - mixed women and men, 18*+*)*

While safe foods was a priority choice for all groups, its selection was not without deliberation. Differences arose in how participants viewed the communitarian value of the intervention and its two components. In deliberation around participants’ experiences with the sale (and purchase) of unsafe and unhealthy food from street vendors, some participants ascribed the consequences to the responsibility of individuals. Hence, a participant claimed the intervention to be *“important but not at the top of the list of important things”* and felt that “*there are more important services here that the community can benefit from*” *(Group 3—older men, 50* +*).*

Those favouring the investment in safe foods outlined the intervention’s community benefit through examples showing how it promotes well-being among particular population groups, notably children.

*“Participant 7: I think when we are here, we are forgetting that we are buying for the community and not for individuals. We are buying for the community; which one out of all these factors is it that important for our community to have?*

*Participant 3: Yes, I do understand that we are buying for the community, but the community also benefits from this food because the same foods are the foods that are being eaten by children at school and at day-care centres, so the schools and the day-cares are in the community, they will benefit the community.” (Group 3 - older men, 50*+*).*

Participants felt strongly about the prevalent problem of food-borne diseases in communities and emphasised the intervention’s benefit to child health. Such deliberation led those who initially voted against safe foods to be convinced otherwise. In reflections on contrasting views, there was also a realisation of how different contexts influence participants' perspectives of priority interventions.

*“Facilitator: What made you change your mind?*

*Participant: The reason that they made. It now made me understand. I think that it is also our understanding when it comes to that; maybe some people will prioritise that some won’t. So, it also depends on the townships that we come from because if you put it as children, I think it should also be prioritised.” (Group 3 - older men, 50*+*)*

For the participant who changed his mind, his support for reconsidering prioritising the intervention was predicated on the understanding that safe foods will help children.

#### Community gardens and clubs

Six groups perceived the solution to improve nutrition in their communities to be in developing community gardens and having monthly discussion clubs around healthy eating and weight management. This intervention was perceived to enhance food security of individuals and the broader community alike through access to fresh vegetables and setting up soup kitchens. The availability of a garden was also seen to enhance financial resilience by generating additional sources of income for those who work in the garden by selling fresh produce.

*“Participant 1: I think community gardens can help everyone. To be able to, if you want to, grow vegetables and sell them to people, to be able to get money and teach children and other older people to do gardening and […] not go out and buy things and be able to get those things in the garden.*

*Participant 2: Okay, also, with those vegetables, they can open a soup kitchen and cook for people who are needy, so it will help a lot.” (Group 4—younger women, 18–49)*

Features of community gardens and clubs, such as nutrition education and skills acquisition, were seen to contribute to personal and social capital, emphasising the value of capabilities for individuals and communities. Participants argued that the intervention would ensure the future utility of certain skills, increase individuals’ and communities’ autonomy, and ultimately reduce dependency on welfare services.

*“I second him because this is very important. Instead of just giving a person food, you are giving them an opportunity to produce food for themselves. So if it is like that, it is a generational wealth that we can have once, and it can continue for ever. So, it will help individuals as well as the community to know that we must do it ourselves and not wait for the handouts.” (Group 5 - mixed women and men, 18*+*)*

Again, participants emphasised the need for community gardens, especially for those struggling with low incomes or job insecurity, highlighting the importance of equity and solidarity considerations when making investment decisions.

*“Participant 2: There are families and orphaned children who do not have food to eat. So, with this community garden, we can go into the community and give those parents food to eat and prevent them from sleeping on an empty stomach because we will be creating a sense of unity.*

*Participant 3: I agree with the decision. Because currently, there are no jobs, and there is no money. So, it would be better for us to farm, and we can sleep with food in our stomachs. Because there are homes with unemployed family members, and what do they eat?” (Group 2 - younger men, 18-49)*

Further reasons for investment included the benefit of social cohesion of community gardens and clubs that would be brought about by working together. Participants believed that gardens and clubs would enable them to meet more frequently, form relations with each other and offer mutual assistance.

*“It should help us to know each other better - as men and women of the community. Women have their things to plant, men have got their things to do - you can build a bigger garden and work together as a community. This thing helps us never to spend money on those street vendors. […] It helps with building friendships, know each other better, respect because you don't live alone in this world. So, I like that one a lot because it brings us together instead of only meeting at stokvels or society meetings only.” (Group 2 - younger men, 18-49)*

Other reasons put forward for investment included a focus on harm prevention. For some participants, regular involvement in gardening and clubs was directly associated with less time spent engaging in unhealthy behaviour like alcohol consumption.

*“As men and women, let us meet at the garden. Let us not only attend society meetings every Sunday. It should not be that every Sunday we would like to go to the drinking pubs/taverns instead of doing something good for our children or ourselves.” (Group 2 - younger men, 18-49)*

#### Food pricing

Apart from two groups, reducing the price of healthy foods was a priority solution to ensure food security and improve nutrition of mothers and children in the communities. The intervention was perceived to *“decrease the levels of hunger that we have in our communities, because if food can be reasonably priced, then the more people can afford nutritious food.” (Group 3—older men, 50* +*).*

Participants reflected on their experiences of healthy food being more expensive than unhealthy. By selecting food pricing, they have foreseen more individuals, especially people experiencing poverty, having increased access to healthy food. The focus on equity was a common consideration that informed participants’ allocation decisions as they felt the less privileged were at a disadvantage.

*“Olive oil is expensive. You cannot afford it. You can only afford fish oil which costs R10 [USD 0.62] from the tuckshop. So, there are people that are not working and people that cannot afford it. So, you must think about that.” (Group 2 - younger men, 18-49)*

Food pricing’s perceived necessity and efficiency as an intervention to remedy existing inadequacies in the current social welfare system were also emphasised. For example, participants in Group 6 traded links to jobs intervention in favour of food pricing. A participant holding this view argued that communities would not be able to realise the full benefit of the originally prioritised links to jobs given current contextual constraints and suggested that the group allocated its limited budget to an intervention with more immediate nutritional benefits.

*“Currently, the reality is that there are no jobs and there is no solution, but in the meantime, the government can maybe regulate food prices since we don’t have a solution for links to jobs.” (Group 6 - mixed women and men, 18*+*)*

Similarly, deliberation in another group revolved around participants’ reflections on the limits of existing social support services in ensuring healthy nutrition and the complementary value of the intervention. Participants perceived that lower food prices would enable the recipients of social support to maximise its intended benefits, enhancing the efficiency of what is already being provided.

*“I would buy food pricing because a lot of people in our communities are getting something from the government. It is just that, that little something is not enough to make them get proper healthy food. So, if food pricing can go down, the minimum of those R350’s or those grants for children can go a long way in the households.” (Group 5 - mixed women and men, 18*+*)*

#### Links to jobs

Lack of regular employment posed challenges for many participants in providing necessities to keep mothers and children well nourished. Participants agreed that work opportunities were limited. Assistance with links to jobs was considered necessary to address the social and economic precursors of poor self-value associated with failure through reducing isolation, earning income and feelings of usefulness.

Several participants spoke about the connection between jobs and children’s well-being. Employment was perceived as necessary to improve caregivers' abilities to accommodate the needs of children by raising family income and resources.

*“I agree with job links because you find that people can’t take care of their children because they don’t have any jobs so that the children can grow and have better support.” (Group 0 - mixed women and men, 18*+*)*

Other benefits associated with job security and consequential poverty reduction included crime prevention.

*“It will help in ending poverty and things like crime because people are pouring out there in the community, and that is why they mug us. There are no jobs. So, I think that can help the community.” (Group 0- mixed women and men, 18*+*)*

For some, investing in links to jobs served more individual purposes than the community. A participant raised this point to his group, thereby challenging the opinions of others while voting against the intervention’s inclusion in the package.

*“I beg to differ. With job links, okay, fine; people; may find jobs, but note that doesn't help the community as such. It helps individuals, whereby now we are looking at something that would help the community, nutrition-wise. […] Finding jobs is good, but is it going to help the community? It's going to help the individual that got the job, but health-wise, it's not going to uplift the community.” (Group 2 - younger men, 18-49)*

The same young man expressed his concern regarding the opportunity costs of choosing links to jobs and being left with less CHAT budget to be spent on another intervention that would be more suitable to improve nutrition in the community.

*“What we are looking at right now is nutrition - most of the time. We are looking at mother and child health. So, it means that somewhere there, we’re going to have a shortage of things that would be more valuable and more important on our chart.”*

#### Low priority interventions

Interventions that received lower priority included a healthy food basket (4 votes), day care (3 votes), and couples antenatal education (2 votes). Nevertheless, these were the subjects of lengthy deliberations.

There was a tendency to dismiss investment in the provision of healthy food baskets to accompany the existing child support grant (ZAR 460, USD 29 per month) aimed at lower-income households to assist parents with the costs of the basic needs of their children. In a context where the existing grant was viewed as inadequate to cover even basic needs such as nutritious food, participants acknowledged that food baskets may increase food security and serve as in investment in children. Despite that the intervention was also considered to improve financial resilience by mitigating food-related economic pressure within the household, three groups decided to spend their money elsewhere. Reasons against investment were guided by participants' lack of trust that the intervention would reach the intended beneficiaries, the children. Their pessimistic perception of the intervention’s potential misuse was mirrored by their experience of parents abusing the grant money and food vouchers they received and spending it on themselves, drugs and alcohol. Participants resisted investing without evidence to guarantee the appropriate use of the intervention.

*“I disagree with this because it's for the child, and there is no guarantee that the child will get it because even now, as we speak, some of the grant money meant for children does not get spent on the things that the child needs. Unless there is a way for us to guarantee that the children will get to eat from the food basket.” (Group 3 - older men, 50*+*)*

Having low-cost or free day care was a priority predominantly for women groups. It was recognised as essential to parental labour force participation and the corresponding financial resilience for families, as well as central to early child development and developing a sense of social cohesion from an early age. On the other hand, there was a tendency to trade off such benefits due to concerns over lack of food safety and poor quality of care in crèches, perceived as a threat to child health and development. The competing priorities and the difficult trade-offs were mirrored by participants' confusion.

*“We had said day care [would help], but now we are confused because the day-care food is making him sick.” (Group 6—mixed women and men, 18 +)*

Participants recognised several benefits of couples antenatal education, including improved gender equity *“so that both parents can assist with taking care of the baby and not just relying on one parent” (Group 0—mixed women and men, 18* +*);* maternal mental well-being *“so the mom doesn’t even have to worry about leaving the child with the father (Group 0—mixed women and men, 18* +*)*; and men’s autonomy for childcare *“because you will find most times it's the mother who is working or busy with something else, so you as the man it's important that you make sure the child is eating the correct food” (Group 3—older men, 50* +*)*. Nevertheless, only two women groups prioritised the intervention. Lack of investment occurred in a context where all groups spent considerable time deliberating the moral obligation of men to look after women and children.

Another common reason for dismissing interventions was the belief that the issue is already being dealt with or unnecessary.

*“If I had an opportunity to change anything, I would change nutrition communication. […] You do not have to go to school to know that. It is important to eat healthy, eh... with that money rather invest it on gardening at schools for children.” (Group 5 - mixed women and men, 18*+*)*

## Discussion

This study aimed to understand the priorities and underlying values of one township community in urban South Africa to improve the nutrition of mothers and children. Our analysis focused on the outcome of the deliberative process of allocating limited nutrition funds, the different groups’ consensus on priorities, and the content of the deliberative discussions. We identified reasons behind the intervention choices and the values driving these choices.

Participants' choices and reasons reflected a tendency to prioritise interventions outside the health sector. Of the 14 intervention options, only 5 were situated within the healthcare sector, representing the lowest priorities for study participants. These included the provision of nutrition supplements to teenage girls and pregnant women, nutrition education campaigns, clinic-based couple antenatal education, and clinic- and home-based nutrition support for mothers. Instead, in the context of high unemployment and financial insecurities, there was a tendency to make choices that would enable communities’ social and economic empowerment and avoid static welfare dependency. Participants preferred interventions that would support children’s optimal development and well-being and interrupt intergenerational cycles of poverty and poor human development in the community. This included prioritising community gardens, having monthly discussion clubs around healthy eating and weight management, and investing in school breakfast to support children in realising their future potential. Deliberations also demonstrated that participants could move beyond self-interest, and at times, change preferences to uphold shared values that considered the communities benefit. Furthermore, there was a tendency to increase efficiency in resource allocation. In this regard, values such as necessity, evidence, and trust were essential to guide participants’ decisions. Decisions not to invest in an intervention were driven by a tendency to assume a pessimistic outcome based on participants’ lived experiences.

Drawing on insights from a novel human development framework by Desmond et al. (2021), investing in early life nutrition interventions without considering barriers to human development over the life course is not sufficient [39]. Complementary interventions are needed that enable communities to realise the potential value of nutrition interventions, an approach of relevance in the Soweto setting where communities face adverse socio-economic environments across their life course. By engaging communities in and analysing the content of deliberations, this study provides unique insights into the contextual constraints of a particular community around nutrition. These insights can inform multi-sectoral investment decisions to enhance the realisation and utilisation of potential nutritional gains aligned with community priorities and values. In turn, the study contributes to the ongoing discussion on the benefit of allowing public deliberation and input to constitute an integral part of public decision-making processes. Targeting contextual barriers through multi-sectoral investments may enhance the value of existing nutrition interventions in the first 1000 days and inform optimal future investments.

Our study also sets out a way to identify more granular values recognised as necessary for achieving overarching values of health systems [40] and other public policy sectors. Researchers investigating the role of values in health decision-making in Latin America emphasised the need to differentiate between what they defined as *core values* of health systems, such as equity, quality, solidarity, universality, and more granular *intermediate* values. The reason is that core values can have different meanings or connotations depending on the perspective of each government [40].

Our study shows that deliberative engagements have the potential to demonstrate such granularity by identifying context-specific intermediate values that ultimately can inform better policy design and more efficient priority setting. For example, equity was a core value in our study concerning improving the nutrition of mothers and children. Some theorists hold that equity can be understood as the general principle of distributive justice and fairness [41, 42], which concerns how individuals and groups are treated relative to one another. Distributive justice has many forms, one being prioritarianism, which strives to improve the condition of the worse off. While specifying prioritarianism as an intermediate value already provides a better understanding of how best to respond to equity needs in each community, there is still a need to understand whom we classify as the most vulnerable or worst off. Different interpretations can lead to different outcomes. Are the worst off those with worse health or those with poorer employment opportunities? If the former should be prioritised, outcomes might exacerbate inequalities if the worst health occurs, for example, among those with higher socioeconomic status.

The deliberations in our study demonstrated that communities prioritised interventions to improve the nutrition of vulnerable groups and provided information on the groups they regarded as such, who for this particular community were those unemployed, with low-income, young first-time mothers, and children.

One approach to integrating social values in priority setting is to leverage standardised healthcare priority setting processes such as health technology assessment (HTA) [43]. While South Africa plans to establish an HTA entity [44], complementary approaches to further consider these values in priority setting processes may be through applying a recently developed South African specific Ethics Framework for health priority setting [45]. The participants’ values described in the present study resonate with some of the 12 domains of the Ethics Framework. Equity, personal financial impact, solidarity and social cohesion, impact on personal relationships and impact on safety and security were 6 of the 12 domains of the Framework that are similar to our findings. If policy makers are to deliver on constitutional values and public sector goals in a meaningful way, public engagement on ethical and social values should be part of an institutionalized priority-setting process. Establishing a South African HTA agency provides a window opportunity for this.

## Limitations

Findings of the study represented the choices of one community. The most important interventions for one community may be less critical for another because of a range of social factors, including politics, culture, social demographics, religion, and levels of economic development. Nevertheless, the present CHAT process adds to the accountability and transparency of any decision-making for mother and child nutrition in the immediate context. The methodology used to modify and implement the exercise could be replicated in a different setting (peri-urban, semi-rural and rural areas) and on a broader scale. It is also not expected that priority interventions will directly translate into policy, particularly without an accommodating local and national policy environment. Translating findings from public engagement into practice warrants further discussions and requires empirical investigations around the uptake of evidence by policy makers. It might be helpful to engage policy makers in CHAT or other deliberative priority setting exercises to establish and compare their priorities with those of the public they represent.

The value elicitation component of the study was based on an indirect approach rather than explicit data collection. Participants were not predisposed to any information on value elicitation before the exercise. The deliberative processes of participants often indirectly, rather than explicitly, communicated value judgements and could be interpreted differently by other researchers. Our strength was that our team included a top bioethicist, and we consulted during the preparatory phase of our work with an independent bioethicist. Community engagement around the identified values could help solidify this work.

## Conclusion

This study demonstrates one approach to public engagement, and the results could have important implications for how we allocate scarce resources to accelerate progress on maternal and child nutrition. Through deliberation, participants could make difficult resource allocation decisions and priorities and underlying values were identified. Further efforts are needed from researchers, funders, and policy makers in multiple sectors to identify and prioritise interventions that communities perceive important in the realisation of optimal mother and child nutrition. Institutionalising public engagement methods through its integration into an HTA agency can help translate the work into practice.

## Supplementary Information

**Additional file 1.** Description of the 14 CHAT interventions as provided to participants.

## Data Availability

The dataset generated and analysed during the current study is not publicly available due to limitations of ethical approval regarding participant confidentiality but are available from the corresponding author upon reasonable request.

## Abbreviations

CHAT: Choosing All Together
HTA: Health Technology Assessment
INPREP: Improved Nutrition Preconception Pregnancy Post-Delivery
WHO: World Health Organization
ZAR: South African Rand
