# Supplementary material for: What values drive communities’ nutrition priorities in a resource constrained urban area in South Africa?
Source: BMC Public Health. 2023 May 12;23:873. doi: 10.1186/s12889-023-15761-1 (PMC10175056; doi:10.1186/s12889-023-15761-1)
Supplement: Supplementary file 1 — Additional file 1. Description of the 14 CHAT interventions as provided to participants. [file 12889_2023_15761_MOESM1_ESM.docx]

**Supplementary information**

*Supplementary Table 1 Description of the 14 CHAT interventions as provided to participants*

| Intervention | *By choosing this…* |
| --- | --- |
| Community gardens and clubs  (1 sticker) | - There will be a vegetable garden in your community. - There will be monthly discussion clubs that will help you eat healthier and manage your weight. |
| Healthy food basket  (25 stickers) | - Children will receive a monthly food basket in addition to the Child Support Grant. The basket will have healthy foods. - Babies and children can eat a healthier diet. - Children will be less hungry and sick. |
| Pregnancy supplements  (1 sticker) | - Pregnant women will receive 13 extra supplements as well as the regular iron and folate supplements in one tablet. This will help prevent premature delivery and low birth weight. - Pregnant women and their babies will become healthier. |
| Food pricing  (1 sticker) | - Healthy foods will reduce in price and cost less. Unhealthy foods with too much oil, sugar, and salt will increase in price and cost more. - You can buy more nutritious food for less money. - You can improve your diet and keep healthy. |
| School breakfast  (2 stickers) | - There will be free breakfast for children at schools every day. This is in addition to the usual government nutrition programme. - The extra food will help children learn better and be healthy. |
| Nutrition education and supplements  (2 stickers) | - Iron and folic acid supplements will be given to teenager girls in schools (because they carry babies). This will prevent girls from getting anaemia (or thin blood). - Children will learn about nutrition *(healthy eating; harms of junk food and sugary drinks; importance of exclusive breastfeeding).* |
| School food garden  (1 sticker) | - There will be food gardens in schools. - Children will learn how to grow vegetables. Promote better nutrition. - The gardens will keep children physically active. |
| Food safety  (2 stickers) | - Street food sellers will learn about food safety and hygiene. The food they prepare will be safe. This will prevent illness from food. - Foods at schools and crèches will be monitored (inspected) to ensure it is fresh and safe to eat. This will protect your children from getting sick from foods. |
| Day care for working parents  (11 stickers) | - There will be low-cost or free day-care for children - Your child (under 6 years) will be looked after while you are at work. - You do not have to miss work. |
| Link to jobs  (1 stickers) | - You will receive help to apply for jobs. - You don’t have to look for jobs alone, a social worker will help. - You might learn about new opportunities. |
| Paid maternity leave  (8 stickers) | - Moms will receive income during maternity leave up to 6 months (formal and informal sector). - This helps lessen the worry of money. - Moms can stay home longer with their babies. They can breastfeed longer. |
| Nutrition communication  (2 stickers) | - You will see information in the media about how to feed your baby (breastfeeding, complementary feeding). - Posters, films, and radio messages will encourage men to be involved in caring for the baby. - Moms will receive regular SMSs (on healthy diet, vitamins, important foods for moms and babies) and clinic reminders. |
| Couple antenatal education  (7 stickers) | - Mothers and their partner will receive education at antenatal clinics. They will learn about:   - nutrition during pregnancy   - how to care for a pregnant woman and a baby   - how to feed babies appropriately. - Men will learn how to support pregnant women and mothers. |
| Mothers nutrition support  (7 stickers) | - Moms will get individual nutrition advice at the clinic (from breastfeeding specialists).   - Learn about healthy diets and healthy weight.   - Why moms should avoid ash or soil, ice, alcohol, tobacco, and drugs. - New moms will be visited 3 times at home by a community health worker.   - Examine the baby and the mom.   - Screen the mom for depression.   - Help moms with any questions about nutrition. - Monthly meetings for pregnant women and moms at the clinic:   - A lady doctor, nutritionist and community health worker will attend.   - Live demonstrations of healthy recipes. |
